# iG6PSnFR: A genetically encoded fluorescent sensor for observing glucose-6-phosphate dynamics in living preparations

**DOI:** 10.21203/rs.3.rs-10883518/v1

**Published:** 2026-10-22

**Authors:** Jonathan S. Marvin, Anastasia Tsives, Shih Ming Huang, Zhanat Koshenov, Raghabendra Adhikari, Aaron D. Wolfe, Ian J. Gonzalez, Daniel Feliciano, Timothy A. Ryan, Matthew J. Merrins, Daniel A. Colón-Ramos, Timothy A. Brown

## Abstract

Glucose-6-phosphate (G6P) is a key intermediate in multiple energetic and anabolic pathways, and quantifying its dynamics is essential for understanding cellular physiology. We have previously developed a syndicate of intensity-based, genetically encoded sensors based on the insertion of circularly permuted GFP into a Venus-flytrap-like analyte-binding protein. Here we use the same approach to develop an intensity-based G6P Sensing Fluorescent Reporter (iG6PSnFR). We present two variants: a G6P-activated sensor that increases fluorescence and a G6P-inactivated sensor that decreases fluorescence. We validate performance across progressively more complex preparations, including purified protein in vitro, immortalized and primary neuronal cultures, isolated pancreatic islets, in an intravital liver model, and finally in vivo in *C. elegans* neurons. In each context, iG6PSnFR reports G6P changes consistent with expected responses to physiological perturbations.

